# Mental health and social service needs for mental health service users in Japan: a cross-sectional survey of client- and staff-perceived needs

**DOI:** 10.1186/s13033-015-0009-7

**Published:** 2015-04-10

**Authors:** Yuki Miyamoto, Rieko Hashimoto-Koichi, Miki Akiyama, Soichi Takamura

**Affiliations:** Department of Psychiatric Nursing, Graduate School of Medicine, The University of Tokyo, 7-3-1 Hongo, Bunkyo, Tokyo 113-0033 Japan; School of Nursing and Nutrition, Shukutoku University, 673 Nitona, Chuo-ku, Chiba, 260-8703 Japan; Division of Nursing, Faculty of Healthcare, Tokyo Healthcare University, 4 Chome-1-17 Higashigotanda, Shinagawa, Tokyo 141-0022 Japan; School of Nursing, Seirei Christopher University, 3453 Mikataharacho, Kita Ward, Hamamatsu, Shizuoka 433-8558 Japan

**Keywords:** Community mental health services, Needs assessment, Health services needs and demand, Shared decision making, Patient-centered care, Schizophrenia, Mental health difficulties, Consumer participation, Housing, Dialogue

## Abstract

**Background:**

The appropriate utilization of community services by people with mental health difficulties is becoming increasingly important in Japan. The aim of the present study was to describe service needs, as perceived by people with mental health difficulties living in the community and their service providers. We analyzed the difference between two necessity ratings using paired data in order to determine implications related to needs assessment for mental health services.

**Methods:**

This cross-sectional study used two self-reported questionnaires, with one questionnaire administered to mental health service users living in the community and another questionnaire to staff members providing services to those users at community service facilities. The study was conducted in psychiatric social rehabilitation facilities for people with mental health difficulties in Japan. The paired client and staff responses rated needs for each kind of mental health and social service independently. The 19 services listed in the questionnaire included counseling and healthcare, housing, renting, daily living, and employment. Overall, 246 individuals with mental health difficulties were asked to participate in this study, and after excluding invalid responses, 188 client-staff response dyads (76.4% of recruited people, 83.6% of people who gave consent) were analyzed in this study. A Wilcoxon matched-pairs signed rank test was used to compare the perceived needs, and weighted and unweighted Kappa statistics were calculated to assess rating agreement within client-staff dyads.

**Results:**

Over 75% of participants in our study, who were people with mental health difficulties living in the community, regarded each type of mental health service as “somewhat necessary,” or “absolutely necessary” to live in their community. Most clients and staff rated healthcare facilities with 24/7 crisis consultation services as necessary. Agreement between client and staff ratings of perceived needs for services was low (Kappa = .02 to .26). Services regarding housing, renting a place to live, and advocacy had the same tendency in that clients perceived a higher need when compared to staff perceptions (p < .01).

**Conclusions:**

It is essential for the service providers to identify the services that each user needs, engage in dialogue, and involve clients in service planning and development.

**Electronic supplementary material:**

The online version of this article (doi:10.1186/s13033-015-0009-7) contains supplementary material, which is available to authorized users.

## Background

One of the principles of health care is that care is provided based on need. As such, performing a needs assessment has become central to the practice of care for people with mental illness. Care providers are required to make a comprehensive assessment of the individual’s needs and develop an appropriate care plan to meet those needs [1]. Despite this practice, unmet needs in mental health care continue to be a serious public health problem [2-5].

According to Bradshaw's influential taxonomy, needs are classified into “normative” and “felt” needs [6]. Normative needs are defined by standards given by an expert or professional, such as a care provider. Felt needs are similar to wants, and in the purview of users. Until the 1990s, the needs assessment was largely done based upon objective markers of need as determined by providers.

Nowadays, however, there are efforts to take into account client viewpoints in the needs assessment. With the increased interest in client perception, studies have assessed needs for care as rated by both clients and care providers and investigated rating agreement [7-13]. Most of the studies that investigated agreement between user and care provider ratings reported that the agreement level of perceived need varied by need domain [7-11,14], and some studies reported users rate more areas of no need compared to their care providers [12,13]. Lasalvia et al.’s longitudinal study reported that better patient-staff agreement on needs for care made a significant contribution to predicting improvement in patient treatment outcomes [15]. In other words, concordance in client-staff assessment seems to lead to positive client outcome. While involving clients in care needs assessment is crucial, it is also important to involve clients in consideration of which services are needed.

Increasingly, there is greater recognition of the importance of decisions to utilize health services made by the service users themselves. Patient-centeredness or shared decision-making has also been crucial in psychiatry [16]. In the current Japanese mental health care setting, the efforts to improve mental health care and promote a shift to patient-centered care are still at an early stage. Moreover, the deinstitutionalization of mental health care has made little progress in Japan. In September 2004, Japan’s Ministry of Health, Labour, and Welfare created a vision for the reform of the mental health care system, which included downsizing of the number of psychiatric beds as one of the primary objectives. However, the number of psychiatric beds has not decreased during this decade [17]. There are, however, effective ways to promote client-centered community care for persons with mental illnesses and it is important to improve how we determine the specific community services necessary for people who have mental health issues. Typically, service providers introduce and provide a portfolio of available services to clients. However, if providers underestimate particular service needs for a client, then that client may not be able to utilize such services.

It is necessary to look at the relation between self-perceived need for treatment and objective clinician assessment of need for treatment [18]. Although Pagura et al. examined and compared help seeking and perceived need [19], there has been no past research examining the difference between self-perceived needs and actual needs for service, as evaluated by service providers from various mental health services, using paired data.

### Aims

The aim of the present study was to describe the need for a service, as perceived by both people with mental health difficulties living in the community and service providers. Furthermore, we analyzed the difference between these two necessity ratings using paired data in order to determine implications related to needs assessment in mental health services.

## Methods

### Design and data collection

This cross-sectional study administered self-reported questionnaires to both people with mental health difficulties living in the community and staff members who are working with these clients in community facilities. The study was performed in psychiatric social rehabilitation facilities in Japan that serve people with a mental-health disorder diagnosis. Psychiatric social rehabilitation facilities in Japan include community centers, such as job training centers, activity centers and facilities where staff promote user recovery in community-based settings. In these facilities, mental health rehabilitation training programs, such as social skills training, job training, and housing accommodations are provided to users. In Japan, only people diagnosed with a psychiatric disorder can use psychiatric social rehabilitation facilities, and users visit their psychiatrists at psychiatric hospitals or clinics. The minimum personnel distribution is designated by regulation, and staff/user ratio varies from 3/19 to 8/20 depending on the type of service.

For the purpose of this study, researchers and experts in psychiatric community care nominated psychiatric rehabilitation facilities that were actively addressing care improvement issues (e.g., reporting about their practice in conferences) and we called 40 facilities in eight prefectures to seek their cooperation in this study. We adopted this method to select the facilities instead of a random sampling method because this study needed extensive cooperation from both service users and service staff, and we felt that active facilities would likely be more collaborative in this kind of research.

We wanted facilities from various parts of Japan to participate in this study and were able to include facilities from six regions out of the total eight regions in Japan. The examinations were conducted from November to December 2004.

There were 1,530 psychiatric social rehabilitation facilities and 20,977 users of these facilities in Japan as of October 1, 2004 [20]. The user ratio of males to females was approximately 2:1 in 2004 [21], and individuals with schizophrenia accounted for 73% to 82% [22] of the users of psychiatric social rehabilitation facilities.

Participants were recruited from psychiatric social rehabilitation facilities for people with mental health difficulties in Japan. Overall, 246 individuals with mental health difficulties were asked to participate in this study, and 225 persons from 39 facilities gave their written informed consent. Staff members who are taking care of the participating clients in the facility were also asked to complete a questionnaire regarding the client.

### Questionnaire

The questionnaire was comprised of two questionnaire booklets for each participating client, one for the client to fill-in and the other for the staff person who was taking care of the client to fill-in.

The client fill-in questionnaire was comprised of questions about a client’s living conditions, employment, self-care behaviors, and current service utilization and needs. The same questionnaire assessed the clients’ awareness of early warning signs, and the results were reported elsewhere [23,24]. The staff fill-in questionnaire was comprised of a fill-in form for client demographic characteristics data that could be obtained from client records, and questions regarding needs in terms of client services. Demographic characteristics included client gender, age, hospitalization, financial support (disability pension, social welfare, or mental health welfare), years from initial onset of symptoms, number of hospital admissions, total length of hospital stay, and diagnosis. The staff questionnaire was comprised of questions about the participating client and no questions regarding the participating staff were included.

### Perceived need for mental health service

Questions about client needs for a mental health service were asked in both the questionnaire provided to the client and on the questionnaire provided to staff. We listed 19 mental health services, which included areas of counseling/consultation and healthcare services (7 services), housing (4 services), support when renting a house (2 services), daily living (4 services), and employment (2 services). This service list was originally developed as part of a larger survey, and listed 19 services nominated by specialists in the psychiatric community care as a list of services provided in Japan for mental health.

Clients were asked whether they felt that any of the 19 services listed in the questionnaire were necessary to facilitate living in the community (unnecessary, somewhat necessary, and absolutely necessary). The questionnaire asked, “What do you think you need to keep living in community? Please select from the services listed below.” Clients were also asked whether they were currently using the services in question. Staff respondents were asked whether any of the 19 services were necessary for the client to keep living in the community (unnecessary, somewhat necessary, and absolutely necessary). The questionnaire asked, “As a service provider, what do you think this client needs to keep living in community? Please select from the services listed below.”

The staff of the participating facilities wrote same ID numbers on paired questionnaires prior to providing the questionnaires to respondents. Both client and staff respondents sealed the completed forms in an envelope individually to maintain confidentiality. ID numbers were then used to match the responses of client and staff. Upon completion, the questionnaires were sent back to the researchers with ID numbers only to maintain confidentiality.

### Ethical considerations

The aims and procedures of this study were approved by the Ethical Committee of the Graduate School of Medicine, The University of Tokyo, Japan. The staff of the participating facilities informed all clients orally and in writing about the study purpose and method. Clients signed a consent form, with the understanding that participation in the study was voluntary, that they could withdraw at any time for any reason, that the researchers would link client and staff data sheets using ID numbers, and that the researchers would not retain identifiable information. Clients gave written consent. We assumed that clients of psychiatric social rehabilitation facilities living in the community had sufficient capacity to provide informed consent and we did not ask for a proxy to assist in consent to participate in this study. Staff respondents indicated consent by responding and mailing back the questionnaires.

### Statistical analysis

Descriptive statistics were used to present basic client information. Perceived needs were coded as the following: unnecessary = 0; somewhat necessary = 1; and absolutely necessary = 2. A Wilcoxon matched-pairs signed rank test was used to compare the needs for each service as perceived by clients and paired-staff (α = .05, 2-tailed). Additionally, the overall percentage of agreement and both weighted and unweighted Kappa statistics were calculated for perceived needs ratings. For the weighted Kappa calculation, we set the lower weights for disagreements that were further apart. Weights were 1, 0.5, and 0 for necessity 0, 1, and 2 apart, respectively.

To illustrate consistency and inconsistency of perceived needs of the service in figures, “somewhat necessary” and “absolutely necessary” were integrated into “necessary.” When a client rated a service as “necessary,” while paired-staff rated the service as “unnecessary,” the responses were considered inconsistent. Similarly, when a client rated a service as “unnecessary,” and the paired-staff rated the service as “necessary,” the responses were also considered inconsistent. Figures show data for clients using the service and clients not using the service when illustrating consistency and inconsistency of the client-staff dyad responses as depicted in percentage bar charts.

All data analyses were conducted using STATA 12.1.

## Results

### Participant characteristics

Among 225 persons who gave written informed consent, one person failed to complete his/her questionnaire, and 36 persons did not answer any of the 19 perceived-needs for service questions. After excluding these 37 responses, the responses of 188 client-staff dyads (76.4% of recruited people, 83.6% of people who gave consent) were analyzed in this study.

Client ages ranged from 21 to 76 years old (M = 43.8 years, SD = 11.9). Table 1 provides the other demographic characteristics of the study participants.

**Table 1:** **Demographic characteristics of client participants [N = 188]**

		**n**	**%**	
Gender	Male	137	72.9	
	Female	47	25.0	
	Not provided	4	2.1	
Age	20-29	23	12.2	
	30-39	51	27.1	
	40-49	45	23.9	
	50-59	49	26.1	
	60-	19	10.1	
	Not provided	1	0.5	
Diagnosis	Schizophrenia	144	76.6	
	Mood disorder	14	7.5	
	Anxiety disorder	5	2.7	
	Epilepsy	5	2.7	
	Other	20	10.6	
Living	With family	66	35.1	
	Group home/Care home	70	37.2	
	Alone	37	19.7	
Receiving Welfare Payment				
	Yes	42	22.3	
	No	136	72.3	
	Not provided	10	5.3	
Receiving Disability Pension				
	Yes	106	91.4	
	No	74	32.7	
	Not provided	8	3.5	
		**Mean**	**SD**	**[min-max]**
Number of hospital admissions^a^		3.0	3.2	[0–19]
Total length of hospital stay (months)^b^		50.7	84.5	[0–504]

^a^n = 183. ^b^n = 179.

### Perceived needs for mental health service usage

Table 2 lists client and staff perceived needs for mental health services usage, and their rating agreement. More than 20% of the clients stated that the listed services were “absolutely necessary” (21.6% to 56.1%). More than half of the clients and staff responded that “health-care facilities with 24/7 crisis consultation” are absolutely necessary. “Financial management support services” were rated as unnecessary by 40% of the clients, and 46.8% of the staff.

**Table 2:** **Client and staff perceived needs for mental health services and rating agreement [Number of pairs = 188]**

			**Client rating** ^**b**^ **%**			**Staff rating** ^**b**^ **%**			**Wilcoxon signed-rank test**		**Unweighted**		**Weighted** ^**c**^	
	**Services**	**n** ^**a**^	**0**	**1**	**2**	**0**	**1**	**2**	**Z**	**p value**	**% Agreement**	**Kappa**	**% Agreement**	**Kappa**
C1	Mental health counselors in municipalities	171	21.1	49.7	29.2	11.1	61.4	27.5	−1.36	.175	46.8	.10	71.4	.16
C2	Counselors in hospitals or clinics	171	10.5	50.9	38.6	4.7	48.5	46.8	−2.38	.017	49.7	.11	73.1	.15
C3	Peer counselors or peer supporters who provide consultation for you	174	16.1	47.7	36.2	2.3	46.0	51.7	−4.48	< .001	46.6	.09	69.3	.10
C4	Health-care facilities with 24/7 crisis consultation	171	2.9	40.9	56.1	3.5	35.1	61.4	−0.92	.358	50.3	.03	73.7	.04
C5	Hospitals or clinics which provide crisis outreach services	173	12.1	45.1	42.8	15.6	61.9	22.5	3.67	< .001	45.7	.10	66.8	.05
C6	24/7 telephone consultation services	179	14.5	41.9	43.6	10.6	56.4	33.0	1.08	0.28	44.7	.09	68.7	.10
C7	Outreach services provided by mental health specialists (psychiatric social workers, nurses)	174	19.5	46.6	33.9	28.7	41.4	29.9	1.85	.065	45.4	.16	68.1	.21
H1	Supportive/ed housing which provides lodging services for respite instead of hospital admission	177	12.4	49.7	37.9	16.4	54.2	29.4	2.09	.037	49.2	.15	71.8	.19
H2	Supportive/ed housing which provide you care when your families cannot take care of you	172	20.4	43.6	36.1	37.2	43.6	19.2	4.76	< .001	41.3	.12	64.5	.15
H3	Supportive/ed housing for a short time period to lessen anxiety of living alone	173	26.0	42.2	31.8	40.5	43.4	16.2	4.07	< .001	39.9	.09	63.0	.11
H4	Supportive/ed housing where staff support you	169	22.5	45.0	32.5	40.2	40.2	19.5	3.95	< .001	42.6	.14	62.7	.11
R1	Joint guarantor (co-signer) agent when renting housing	173	15.0	41.6	43.4	38.2	37.0	24.9	5.36	< .001	38.7	.10	63.0	.16
R2	Housing information	168	20.2	45.2	34.5	34.5	44.6	20.8	3.75	< .001	38.7	.07	64.0	.12
D1	Community support center that provides information you need and place to interact with friends	175	9.1	45.1	45.7	0.6	45.1	54.3	−2.94	.003	58.9	.25	77.7	.26
D2	Home help services that help with household tasks such as cleaning, cooking, etc.	171	27.5	43.9	28.7	33.9	39.8	26.3	1.24	.217	43.9	.15	66.4	.19
D3	Advocacy services that listens to and advocates your concerns and complaints about medical welfare	174	22.4	40.2	37.4	25.3	55.8	19.0	2.83	.005	36.2	.02	62.4	.04
D4	Financial management support services which help with your money management	171	40.4	38.0	21.6	46.8	35.7	17.5	1.53	.126	46.2	.16	69.6	.25
E1	Vocational services including job counseling and search	176	9.7	46.0	44.3	12.5	40.3	47.2	−0.17	.867	42.6	.03	68.8	.10
E2	Sheltered workshops where there is someone available to you for consultation	175	13.1	40.6	46.3	12.6	49.7	37.7	1.46	.143	44.0	.08	69.1	.14

^a^Number of pairs of client and staff who both answered the perceived need for the service item.

^b^0 = unnecessary; 1 = somewhat necessary; 2 = absolutely necessary.

^c^Weights were 1, 0.5, 0 for categories 0, 1, and 2 apart, respectively.

C: Counseling and healthcare; H: Housing; R: Renting; D: Daily living; E: Employment.

In terms of client and staff agreement, client and staff rated 11 out of 19 services significantly differently. While clients perceived “Joint guarantor (co-signer) agent when renting housing” as necessary, staff perceived this service as less necessary (Z = 5.36, p < .001). Clients rated services regarding housing, renting a place to live, and advocacy as more important compared to staff. Staff respondents rated counselors in hospitals or clinics, peer counselors, and community support centers significantly higher compared to their clients.

All unweighted Kappa and weighted Kappa coefficients ranged from 0.02 to 0.26.

### Consistency and inconsistency for perceived needs

**Figure 1:**
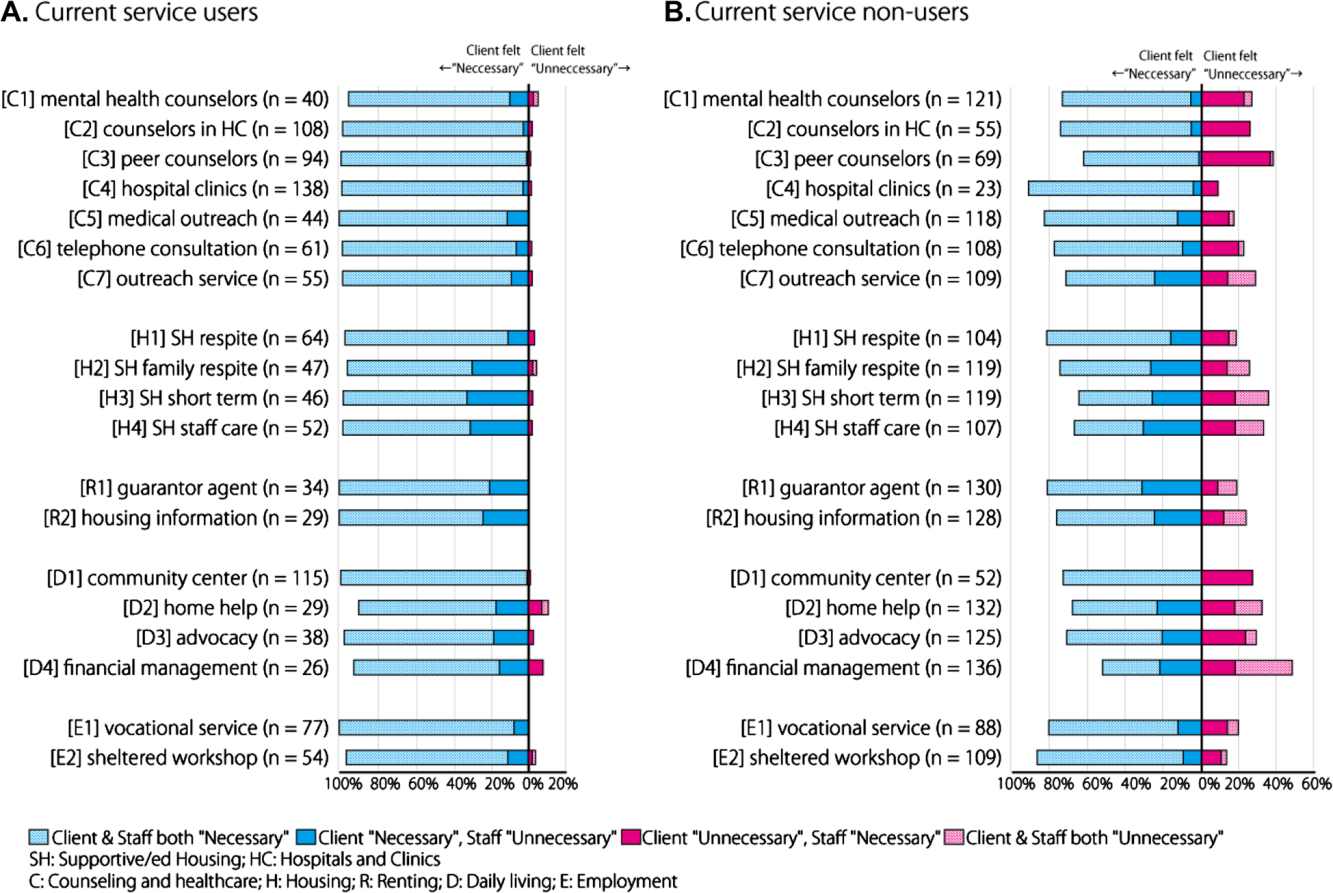
**Agreement between client and staff dyads in perceived needs by actual use of each service.** Figure 1A and B provide the percentages of consistency and inconsistency between client and staff pairs in perceived needs by actual use of each service. **A**, currently using the service; **B**, not using the service. “Absolutely necessary” and “somewhat necessary” were integrated into “necessary.

Figures 1A and 1B provide the percentages of consistency and inconsistency between client and staff pairs in perceived needs by actual use of each service. “Absolutely necessary” and “somewhat necessary” were integrated into “necessary.”

As shown in Figure 1A, most clients who are currently using the service in question rated the service as “necessary,” while some staff perceived the service as “unnecessary” for their clients. This inconsistency was seen especially in housing services.

On the other hand, for clients who are not using that particular service in question (Figure 1B), both types of inconsistency (user rating the service “necessary,” while staff rating “unnecessary,” and user rating the service “unnecessary,” while staff rating “necessary”) were present. Staff were more likely to rate consultation and healthcare services as “necessary,” whereas clients rated this as “unnecessary,” rather than the other way around. On the contrary, clients were more likely to rate housing and renting as “necessary” and staff rated this as “unnecessary,” rather than the other way around. Numerical data of Figure 1 is shown in Additional file 1 (Appendix table).

## Discussion

People with mental health difficulties living in community who are clients of psychiatric social rehabilitation facilities in Japan participated in this research. Nearly three quarters of the clients who participated in the study reported services listed in this research as “absolutely necessary” or “somewhat necessary.” The findings indicated that agreement between client and staff ratings of perceived needs for services was low. The percentage of inconsistency for housing services was relatively high, with clients rated housing services as more necessary compared to staff.

Most clients and staff respondents rated healthcare facilities with 24/7 crisis consultation services as necessary, while nearly 60% of both clients and staff respondents rated them as “absolutely necessary.” Mental health service models, such as the Trieste model [25] and the Assertive Community Treatment (ACT) model, adopt crisis consultation services that operate 24 hours a day, 7 days a week. These models are aimed at recovery and to reduce hospitalization in people with mental health difficulties. In Japan, ACT services were implemented in 2003, and have reduced in-patient days of people with mental health difficulties [26]. Based on such evidence and the perceived needs of clients in our research, these 24/7 services seem to be essential to living in the community.

Agreement between client and staff in the necessity for a client’s service usage was not high, and Kappa and unweighted Kappa statistics were low (0.26 or lower) for all of the included services. In studies that compared patient perceptions of care needs with those of their professional caregivers, Kappa ranged from 0 to 0.67 [9], 0.07 to 0.51 [10], 0.15 to 0.60 [12], 0.20 to 0.95 [13], and 0.33 to 0.84 [14]. In our research, we asked whether clients needed a service to live in the community without asking them whether their needs are met. We cannot compare our results directly with studies that asked about care needs, but we can say that the low agreement rate in our research showed inconsistency in the perceptions of needs for services between clients and staff.

A signed-rank test showed that disagreement between client and staff differed across services. Clients rated housing services (all related to supported housing), services for renting a house (joint guarantor and housing information), and advocacy services as more necessary compared to staff. Housing is a basic human right and a base for community living. Housing interventions, such as Housing First, have been implemented and showed greater community integration, improvements in quality of life, and reduction in mental illness symptoms [27]. Clients in our research were already living in the community, which might be the reason that the staff did not feel there was as much necessity for their clients to use these housing services or receive rent support. The results of our research indicate that it is necessary to ask and listen to client needs, which is directly connected to advocacy.

Furthermore, staff respondents were more likely to indicate that hospital counselors, peer counselors, and community support centers are necessary for their clients. This does not mean that the clients perceived low needs to use these services; instead, it indicated that more than 95% of staff respondents answered that these services are “necessary,” which might have contributed to significant differences between client and staff ratings.

Because we did not obtain demographic information from staff respondents regarding their gender, age, and profession, we could not assess what kind of factors would contribute to the concordance of needs assessment of client and staff. Staff profession, such as being a nurse or social worker might have affected the needs assessment across services because care approach and assessment focus might vary according to profession. Additionally, we did not obtain information about the relationship between the participating client and the staff who completed the questionnaire. For example, we did not ask about the closeness of their relationship, or frequency of conversations, which may have a potential impact on agreement in needs assessment.

Most clients who are using a service rated the service as “necessary.” It is natural that they are using the service because they need the service, and after experiencing the service, users might become aware of the benefits of the service, strengthening their perception that they need the service they are using.

Overall, participants in our study, who were people with mental health difficulties living in a community, regarded community mental health services as “somewhat necessary,” or “absolutely necessary.” However, agreement between the client and staff dyad regarding the necessity of a service was not high. Recently, the importance of shared decision-making has been emphasized, and also emphasized in the community mental health care [28]. Shared decision-making focuses mainly on treatment and care, but this concept can also be adopted in the selection of services. Again, it is necessary to ask clients what services they need instead of obtaining this information from providers. Additionally, when planning and developing mental health services, policy makers need to engage in dialogue with the clients of these services.

Our current study has several limitations. First, we did not obtain any demographic information about staff respondents, and the closeness of their relationship with a client. Ratings and agreement of necessity to use specific services might have differed according to staff profession and relationship with client. However, staff respondents were staff members working in facilities that the participating clients were using, and the reality is, regardless of the depth of the relationship with client, staff members are the ones who introduce service options to clients. Second, this study did not consider the severity of illness or social function level. The necessity of service use could be different depending on these states, and the proportion of each rating of necessity might be different among other groups of people. However, each client-staff dyad assessed the needs for one person, and for the agreement statistics, we adopted matched pairs tests, such as Wilcoxon signed-rank test and Kappa statistics. Third, this study was implemented in selected facilities that were nominated based on being actively-engaged in care improvements in the mental health welfare area. As such, the agreement rate for mental health service users and staff might be lower in the general population. We have to be cautious about the generalizability of our findings because our participants might not be a representative sample.

## Conclusions

Over 75% of participants in our study who are people with mental health difficulties living in the community regarded each community mental health service as “somewhat necessary” or “absolutely necessary.” Agreement between client and staff rating of perceived needs for services was not high. The inconsistency between client and staff ratings in perceived needs was relatively high for housing services, with clients being more likely to feel that these services are necessary compared to staff. As such, it is essential for the service providers and policy makers to ask clients what services they need, communicate with them, and involve them in service planning and development.

## Additional file

Additional file 1:
**Agreement between client and staff dyads in perceived needs by actual use of each service.**
