# Supplementary material for: Mental health and social service needs for mental health service users in Japan: a cross-sectional survey of client- and staff-perceived needs
Source: Int J Ment Health Syst. 2015 Apr 10;9:19. doi: 10.1186/s13033-015-0009-7 (PMC4419408; doi:10.1186/s13033-015-0009-7)
Supplement: Additional file 1: — Agreement between client and staff dyads in perceived needs by actual use of each service. [file 13033_2015_9_MOESM1_ESM.docx]

## Additional file 1: Agreement between client and staff dyads in perceived needs by actual use of each service

|  |  | Current service users | | | | |  | Current service non-users | | | | |
| --- | --- | --- | --- | --- | --- | --- | --- | --- | --- | --- | --- | --- |
|  | Services | n | Client & Staff both "Necessary" | Client "Necessary", Staff "Unnecessary" | Client "Unnecessary", Staff "Necessary" | Client & Staff both "Unnecessary |  | n | Client & Staff both "Necessary" | Client "Necessary", Staff "Unnecessary" | Client "Unnecessary", Staff "Necessary" | Client & Staff both "Unnecessary |
|  |  |  | % | % | % | % |  |  | % | % | % | % |
| C1 | Mental health counselors in municipalities | 40 | 85.0 | 10.0 | 2.5 | 2.5 |  | 121 | 67.8 | 5.8 | 22.3 | 4.1 |
| C2 | Counselors in hospitals or clinics | 108 | 95.4 | 2.8 | 1.9 | 0.0 |  | 55 | 69.1 | 5.5 | 25.5 | 0.0 |
| C3 | Peer counselors or peer supporters who provide you with consultation | 94 | 97.9 | 1.1 | 1.1 | 0.0 |  | 69 | 60.9 | 1.5 | 36.2 | 1.5 |
| C4 | Health-care facilities with 24/7 crisis consultation | 138 | 95.7 | 2.9 | 1.5 | 0.0 |  | 23 | 87.0 | 4.4 | 8.7 | 0.0 |
| C5 | Hospitals or clinics which provide crisis outreach services | 44 | 88.6 | 11.4 | 0.0 | 0.0 |  | 118 | 70.3 | 12.7 | 14.4 | 2.5 |
| C6 | 24/7 telephone consultation services | 61 | 91.8 | 6.6 | 1.6 | 0.0 |  | 108 | 67.6 | 10.2 | 19.4 | 2.8 |
| C7 | Outreach services provided by mental health specialists (psychiatric social workers, nurses) | 55 | 89.1 | 9.1 | 1.8 | 0.0 |  | 109 | 46.8 | 24.8 | 13.8 | 14.7 |
| H1 | Supportive/ed housing which provides lodging services for respite instead of hospital admission | 64 | 85.9 | 10.9 | 3.1 | 0.0 |  | 104 | 65.4 | 16.4 | 14.4 | 3.9 |
| H2 | Supportive/ed housing which provide you care when your families cannot take care of you | 47 | 66.0 | 29.8 | 2.1 | 2.1 |  | 119 | 47.9 | 26.9 | 13.5 | 11.8 |
| H3 | Supportive/ed housing for a short time period to lessen anxiety of living alone | 46 | 65.2 | 32.6 | 2.2 | 0.0 |  | 119 | 38.7 | 26.1 | 17.7 | 17.7 |
| H4 | Supportive/ed housing where staff support you | 52 | 67.3 | 30.8 | 1.9 | 0.0 |  | 107 | 36.5 | 30.8 | 17.8 | 15.0 |
| R1 | Joint guarantor (co-signer) agent when renting housing | 34 | 79.4 | 20.6 | 0.0 | 0.0 |  | 130 | 50.0 | 31.5 | 8.5 | 10.0 |
| R2 | Housing information | 29 | 75.9 | 24.1 | 0.0 | 0.0 |  | 128 | 51.6 | 25.0 | 11.7 | 11.7 |
| D1 | Community support center that provides information you need and place to interact with friends | 115 | 98.3 | 0.9 | 0.9 | 0.0 |  | 52 | 73.1 | 0.0 | 26.9 | 0.0 |
| D2 | Home help services that help with household tasks such as cleaning, cooking, etc. | 29 | 72.4 | 17.2 | 6.9 | 3.5 |  | 132 | 44.7 | 23.5 | 17.4 | 14.4 |
| D3 | Advocacy services that listens to and advocate your concerns and complaints about medical welfare | 38 | 79.0 | 18.4 | 2.6 | 0.0 |  | 125 | 50.4 | 20.8 | 23.2 | 5.6 |
| D4 | Financial management support services which help with money management | 26 | 76.9 | 15.4 | 7.7 | 0.0 |  | 136 | 30.2 | 22.1 | 17.7 | 30.2 |
| E1 | Vocational services including job counseling and search | 77 | 92.2 | 7.8 | 0.0 | 0.0 |  | 88 | 68.2 | 12.5 | 13.6 | 5.7 |
| E2 | Sheltered workshops where there is someone available to you for consultation | 54 | 85.2 | 11.1 | 1.9 | 1.9 |  | 109 | 77.1 | 9.7 | 10.3 | 2.9 |

C: Counseling and healthcare; H: Housing; R: Renting; D: Daily living; E: Employment
